# Specific Intensity Direct Current (DC) Electric Field Improves Neural Stem Cell Migration and Enhances Differentiation towards βIII-Tubulin+ Neurons

**DOI:** 10.1371/journal.pone.0129625

**Published:** 2015-06-11

**Authors:** Huiping Zhao, Amanda Steiger, Mitch Nohner, Hui Ye

**Affiliations:** Departments of Biology, Loyola University Chicago, Chicago, Illinois, United States of America; Temple University School of Medicine, UNITED STATES

## Abstract

Control of stem cell migration and differentiation is vital for efficient stem cell therapy. Literature reporting electric field–guided migration and differentiation is emerging. However, it is unknown if a field that causes cell migration is also capable of guiding cell differentiation—and the mechanisms for these processes remain unclear. Here, we report that a 115 V/m direct current (DC) electric field can induce directional migration of neural precursor cells (NPCs). Whole cell patching revealed that the cell membrane depolarized in the electric field, and buffering of extracellular calcium via EGTA prevented cell migration under these conditions. Immunocytochemical staining indicated that the same electric intensity could also be used to enhance differentiation and increase the percentage of cell differentiation into neurons, but not astrocytes and oligodendrocytes. The results indicate that DC electric field of this specific intensity is capable of promoting cell directional migration and orchestrating functional differentiation, suggestively mediated by calcium influx during DC field exposure.

## Introduction

The adult human brain contains several regions capable of producing neuronal stem/progenitor cells, including the forebrain’s anterior subventricular zone (SVZ) and hippocampus. These areas provide valuable resources for neural regeneration. In a pathological condition such as cerebral ischemia, stem cells migrate to the injured brain area for repair [1–5]. However, only a very small portion of the newly generated NPCs are ultimately found to migrate to the targeted areas and become functional cells [2, 5, 6]. Unlike most organs in the human body, the capability for the brain to regenerate is very limited.

To compensate for the limited availability of stem cells for neurogenesis, laboratory studies are now focusing on direct transplantation of cultured adult NPCs into the injured area. Although this approach has been reported successful in promoting the formation of new nerve cells, it is generally accepted that transplanted cells experience great difficulty migrating and regenerating neurons inside the injured tissue [7–9]. Our current understanding of stem cell migration and differentiation concentrates on inducing factors through cytokine-mediated biochemical signaling that would activate cell surface receptors and trigger signal cascades, thus, resulting in activation of intracellular pathways that promote cytoskeletal reorganization and subsequent migration [10–12]. Identification of these molecular mediators and adult neurogenesis remains a daunting task in current research.

Taking a bioengineering approach, several works have reported that electric fields can be used to stimulate and direct the migration (termed galvanotaxis) of neural stem cells *in vitro* or *ex vivo* [13–17]. These experiments are based upon the understanding that endogenous electrical signals are present in many developing systems [18], and that crucial cellular behaviors are under the influence of such endogenous electric cues including: cell division, migration, and differentiation. Intensity of the electric fields must be appropriately controlled to induce cell migration without introducing damage. Although publications describing the movement of cells under the influence of an externally-applied electric field can be retrieved from the 1920’s [19], the underlying mechanism of the electric field’s action is largely elusive.

In conjunction with migration studies, electric fields have also shown their potential in guiding various stem cells into the neuronal lineage. An intermittent and systematic DC electric stimuli can guide human mesenchymal stem cells (hMSCs) towards neural-like cells [20] with minimal cellular damage. In contrast, alternating electric current (AC) [21], or pulsed electric field combined with an optimized biochemical microenvironment [22], introduced osteogenic differentiation of hMSCs. In another example, monophasic and biphasic pulsed electric fields were applied to the human cardiac progenitor cells (hCPCs) isolated from human heart fragment, and induced early differentiation towards a cardiac phenotype. Interestingly, only the biphasic fields showed effectiveness in the up-regulation of cardiac transcription factors [23]. Within the same AC electric field, cell differentiation could be a function of the field frequency. Osteogenic differentiation of human adipose-derived stem cells depended on the frequency of the applied electromagnetic field, with 30 Hz and 45 Hz favoring the osteogenic differentiation [24]. Therefore, properties of the electric field played significant roles in fine-tuning and guiding these stem cells into neuronal lineages.

Electric field has also demonstrated potential in promoting neural stem cell differentiation toward neurons and their enhanced maturation. Short duration electrical stimulation at physiological level (0.53 or 1.83 V/m) was effective in enhancing neurite outgrowth and maturation of adult neural stem progenitor cells [25]. Ariza et al [26] found that the NPCs treated with a 437 V/m direct current (DC) EF aligned perpendicularly to the field vector had a greater tendency to differentiate into neurons, but not into oligodendrocytes or astrocytes, compared to controls. However, the mechanism of such action has not been elucidated, and the cells have a decreased cell viability under this specific field intensity. Optimal control of various field parameters appears to be the key in safe, electrically-guided neural stem cell migration and neuronal differentiation.

The abilities of stem cells to migrate into the target area, differentiate into the desired cell types, and functionally integrate with the existing tissue are the ultimate goals for stem cell therapy. A single electric field that can provide simultaneous control of both cell migration and desired differentiation would hold significant potential for stem cell therapy. In this paper, we report that 115V/m DC electric field can induce neural stem cell migration *in vitro* in a calcium-dependent manner. In addition, this low field intensity shows a capability to drive differentiation of NPCs into neurons.

## Materials and Methods

### 1. Ethics statement

All experimental protocols in this study were approved by the animal care committee of the Loyola University Chicago (#27), in accordance with the policies established by Institutional Animal Care and Use Committee (IACUC).

A total of 23 young C57BL/6J mice (P2-7, Charles River Laboratories, Wilmington, MA) were used in this study. Mice were kept in the Loyola University Chicago Animal Facility under continuous care by facility technicians.

### 2. Isolation and culturing of neural stem cells

Neural precursor cells (NPCs) were obtained as described previously [27]. Briefly, C57BL/6J mice (P2-7) were treated with isoflurane, and sacrificed by cervical dislocation. Brains were dissected, and the periventricular region was cut into small pieces (approximately 1.0 *mm* ^3^) and enzymatically dissociated. Cells were plated at 10–100 cells /μl in T25 or T75 culture flasks (BD Falcon) in Serum Free Medium Completed (SFMC) that was supplemented with epidermal growth factor (EGF, 20 ng/mL; Sigma-Aldrich), fibroblast growth factor (bFGF, 10 ng/mL; Sigma-Aldrich) and heparin (0.73U/mL; Sigma-Aldrich). Cells were incubated with 5% CO_2_ at 37°C and 100% humidity and refreshed with SFMC medium every 2–3 days. After 7 to 10 days, primary neurospheres formed, consisting purely of Nestin positive NPCs. Cells were passaged once every week. Primary neurospheres were utilized for passaging to next generation. Cells collected from passage 3 and 4 were used for cell differentiation assay, whole cell patching experiment, and migration analysis.

### 3. Live cell imaging and kinematic analysis of undifferentiated NPCs

To generate NPC slide samples for electric field exposure, 10 mm x 10 mm coverslips (Platinum Line, #1) were UV-sterilized and incubated in poly-D-lysine at room temperature for 2 hours. The slides were transferred into 24-well cell culture plate (BD Falcon) and washed with 0.22 μm filtered H_2_O. The slides were then coated with 200 μL of 95% SFMC / 5% Matrigel (Corning Matrigel Matrix, #354230) to mimic three-dimensional matrix of *in vivo* conditions. After half hour of 37°C incubation of the 5% Matrigel–coated slides, ~ 30,000 NPCs were plated onto each individual slide and 0.4 ml of SFMC was added to each well. The wells were incubated with 5% CO_2_ at 37°C and 100% humidity. Newly-generated NPC slides incubated for 48–96 hours were used for electric field (EF) exposure. The slides were then placed into a galvanotaxis chamber that was capable of generating a DC electric field.

The field was generated by applying a 1.5 V voltage across two parallel Ag/AgCl wires within the chamber. The Ag/AgCl electrodes were made by immersion of the Ag wire (World Precision Instruments, 1.0 mm in diameter) into the Javex bleach overnight. The Power supplier for the 1.5V DC electric field for the cell migration chamber is an Enercell High-Power AC Adapter (Radioshack.com). Electric field strength was calculated by E = V/d, where E is the field intensity, V = 1.5 V and d = 13 mm is the distance between the two Ag/AgCl bars. This generated a field of approximately 115 V/m (1.5 V/13 mm). Intensity of the field was further measured and confirmed (see next section).

Live cell imaging under electric field exposure was performed on an Olympus IX2 inverted microscope combined with a UIS2 optical system, plus a microscope-stage automatic thermo control system. The frame capture speed was 1 frame/min. Migration of cells was analyzed using MetaMorph Microscopy Automation and Image Analysis Software. To investigate the effect of calcium buffering on cell migration in the DC electric filed, 1mM of EGTA (Ethylene glycol-bis(2-aminoethylether)-N,N,N',N'-tetraacetic acid, Sigma-Aldrich) was added to each trial just prior to EF application and the start of live-cell imaging. The length of each of our live cell imaging experiment ranges from 1.5–2 hours. In order to average the data and make quantitative comparison possible, we only analyzed the cell kinematics at 1.5 after E-field treatment in each experiment. If the video was longer than 1.5 hours, the rest of the video after this time point is not included in the analysis. Cells after this experiment were not used for other purposes.

**Fig 1:**
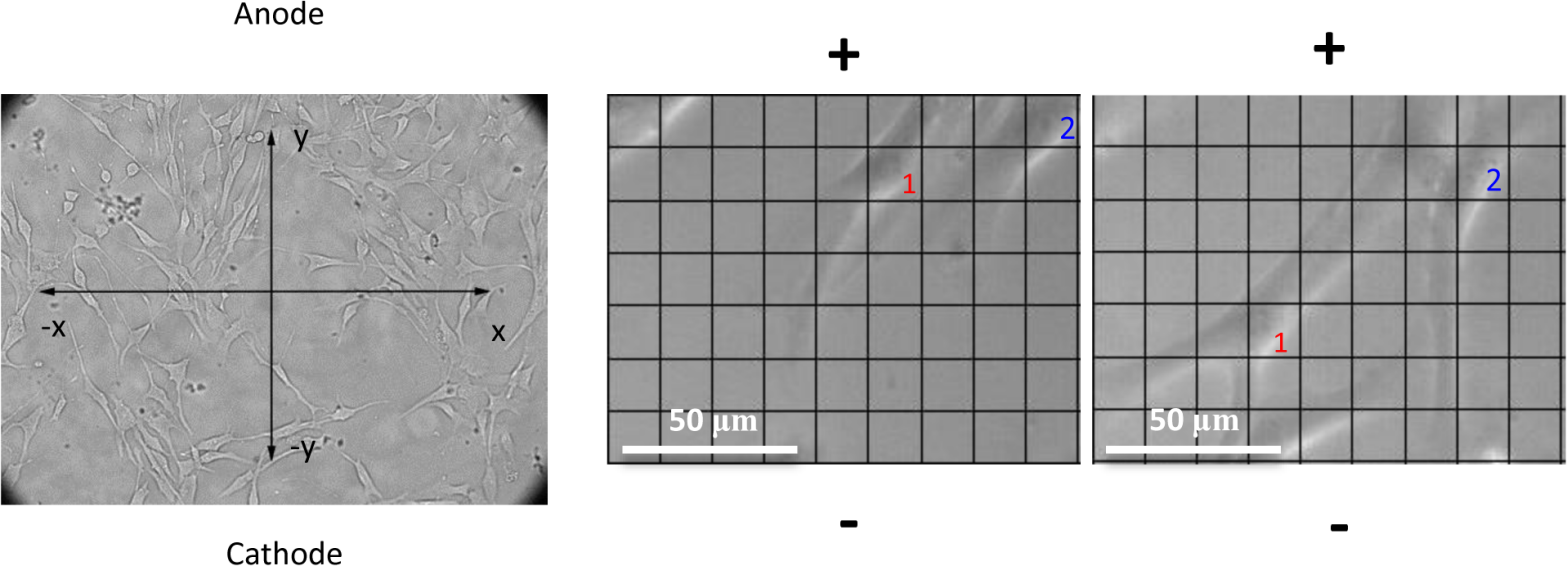
Live cell imaging and grid measurement method to characterize cell migration kinematics. Using a 5mm x 5mm (converted to 15 μm X 15 μm actual size) imposed transparency grid, individual cell movement was analyzed in the electric field. Cells were directed to move towards the cathode (-) under 115 V/m DC electric field stimulation.

The first image from each trial was used to label individual cells. A grid system was mounted to the first frame of each movie and implemented to determine the minimum cell movement required for analysis (Fig 1). All cells that moved one grid cell away from their point of origin were analyzed for distance. The final location of the majority of cell body was marked as the ending point of cell movement (x_end, y_end) and was analyzed with the starting point (x_start, y_start) under the same criteria. The grid size is 15 μm by 15 μm. These methods were used to normalize all cells to a common origin of (0, 0) and to scrutinize the precise movement of each individual cell. The distance travelled in the X and Y directions by each cell was measured.

### 4. Whole cell patching of undifferentiated NPCs in the electric field

**Fig 2:**
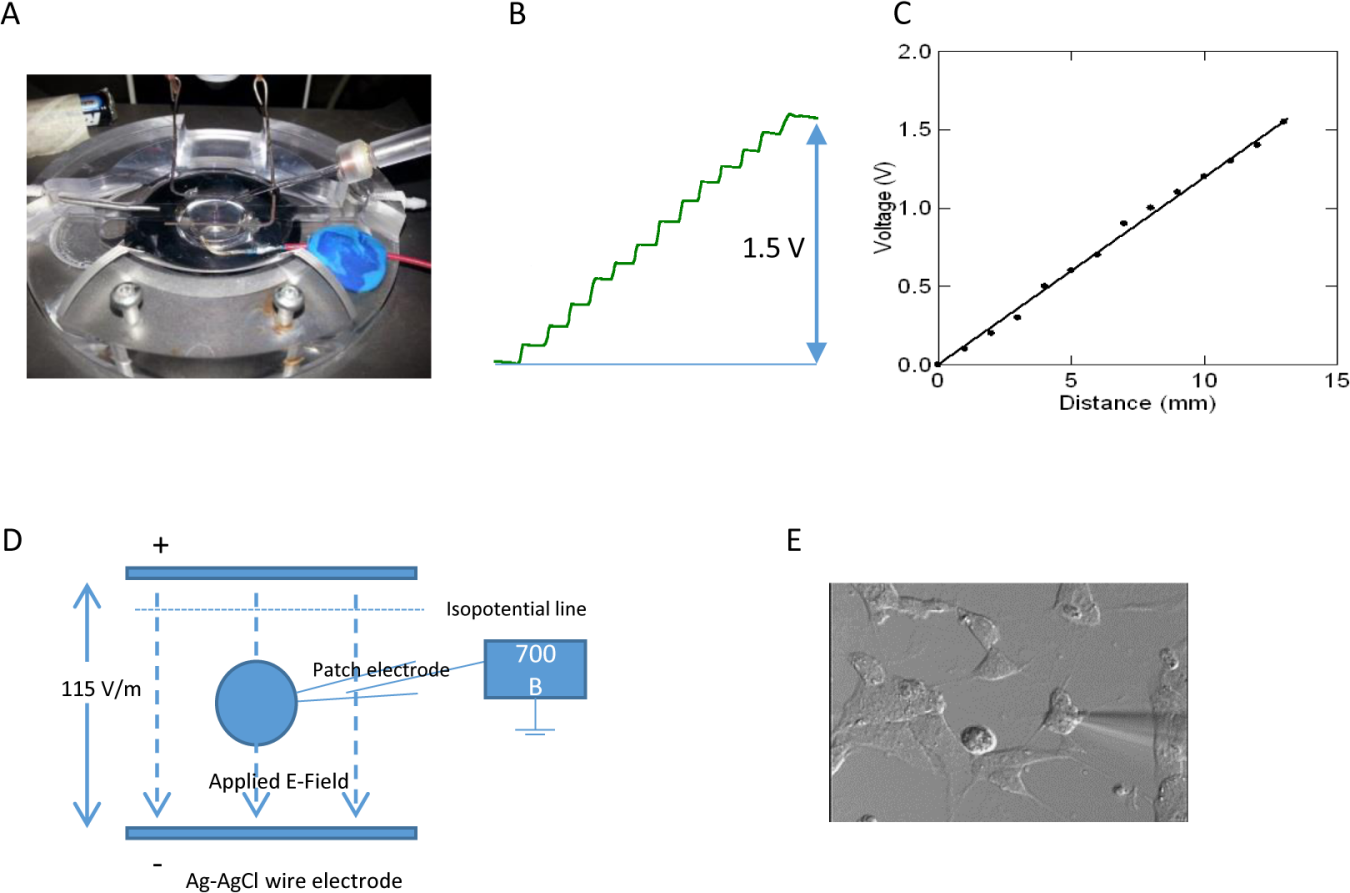
Whole-cell patch clamp recording of the cultured neural stem cell in 115 V/m DC electric field. (A) Experimental setup, including perfusion chamber, two Ag/AgCl electrodes, a 1.5 V battery, and a recording glass electrode. Uniformed electric field was generated by passing electric current between two parallel Ag/AgCl electrodes. Potential difference was generated by the 1.5V battery. (B) Electric potential was measured by moving the electrode from one Ag/AgCl wire to the other one with a step of 1 mm. (C) Plot of voltage change vs. the distance of electrode moved. The field intensity is 115 V/m. (D) For whole cell patching, cells were plated on the matrigel on a small coverslip (10 mm X 10 mm), which was positioned in the recording chamber. Signal was amplified by a 700B amplifier and recorded by a computer. (E) Whole cell patch electrode was applied to the cell under visual guidance.

To record the membrane potential change in the DC electric field, two parallel Ag/AgCl wires were glued in parallel with a distance of 13 mm on the bottom of the recording chamber and connected to a 1.5 V, AA alkaline battery (rayovac.com)(Fig 2A). This produced a DC electric field of 115 V/m. Intensity of the electric field is measured with an electrode moving perpendicularly to the Ag/AgCl wires (Fig 2B) between two points. Using a 1.5 V battery instead of an AC/DC transformer greatly reduced the 50/60 Hz noise during the patch clamp experiment. The potential differences between the two points were recorded as Δ*V* and the distance of the two points was Δ*d*. The field intensity E is calculated by $\frac{\Delta V}{\Delta d}$ (Fig 2C).

Single neural stem cells were seeded on a small coverslip (10 mm X 10 mm in size) and cultured for 3–5 days. For patch clamp recording (Fig 2D), the coverslips were transferred to a submerged recording chamber to be continuously perfused with aerated artificial cerebrospinal fluid (ACSF) at a speed of 4 to 6 mL/min at room temperature. The recording chamber was mounted on a Nikon FN-1 Upright Physiology Microscope (Nikon Instruments Inc, Melville, NY). We used infrared differential interference contrast microscopy to visualize individual cells (Fig 2E). Patch-clamp electrodes were positioned onto the cell membrane under visual guidance using a micromanipulator system (MP-285, Sutter Instrument Company, Novato, CA). Whole-cell recordings were performed using a MultiClamp 700B current & patch clamp amplifier with a CV-7B headstage (Axon Instruments, Foster city, CA, USA). Digitization was performed by a 12-bit A/D board (Digidata 1440A; Axon Instruments), and recorded using pCLAMP software (v 10). The sampling rate was 25,000Hz.

The components of the patch pipette (intracellular) solution were the following (in mM): 120 potassium gluconate, 20 KCl, 2 MgCl_2_, 0.2 EGTA, 10 HEPES, 2 Na_2_-ATP (pH 7.3). Patch pipettes were pulled from borosilicate capillary tubing (World Precision Instruments, Sarasota, FL, USA) with a micropipette puller (Sutter). In some experiments, the pipette solution was supplemented with 1 mg/ml Lucifer Yellow. Electrodes had tip resistance ranging from 4 to 6 MΩ when filled with internal solution. The resistance to ground of the whole-cell seal was 2 to 4 GΩ before breaking through the membrane, and the series resistance was less than 20 MΩ. Whole cell compensation was applied after breaking through the cell membrane. Cells were recorded with the whole-cell configuration in both the current-clamp mode and voltage-clamp mode. A cell was considered acceptable if it had a stable resting membrane potential of at least -50mV. In some voltage clamp experiments, sodium channel blocker tetrodotoxin (TTX, 1.0 μm) and non-selective voltage-dependent calcium channel (VDCC) blocker CdCl_2_ (250 μM) were added to the perfusion solution ACSF.

After the cell was patched, constant 0.025 nA current steps, 500 ms in duration, were injected into the cell to obtain a current–voltage curve, to measure input resistance and capacitance. The slope of the current—voltage plot represented the input resistance. Membrane capacitance was measured by curve fitting for the initial phase of membrane hyperpolarization when negative current was injected. For voltage clamp, cells were held at different step voltage, ranging from -100 mV to 40 mV with 10 mV increments. Capacitive transients were compensated on-line using clamp-amplifier setting.

When a cell was patched on, membrane potentials with and without the presence of DC electric field were measured and compared to estimate the effect of DC electric field on the cell membrane potential (∆Vm). Since electric field also causes voltage change on the recording electric per se, this voltage change has to be compensated. When the whole cell recording was over, the recording electrode was gently withdrawn from the cell membrane, and was positioned on the same isopotential line defined by the parallel Ag/AgCl wires. The measured field potential was further subtracted from ∆Vm to yield a true measurement of change in the membrane potential [28]. To avoid potential damage of the cell by the patching pipette due to cell migration inside the electric field, each patching experiment took less than 5 minutes.

### 5. Differentiation analysis

Chamber Slides (Thermo Scientific, Nunc Lab-Tek) were cut by size 6mm X 6 mm and UV sterilized for 15 minutes. The slides were coated with 100 μg/mL poly-L-lysine (Sigma-Aldrich, Canada) for 2 hours at room temperature, rinsed 3 times with 1 mL of 0.22 um filtered water, transferred into a 24-well cell culture plate (BD Falcon), and then coated with 5% (v/v) matrigel (BD Biosciences) in SFMC for half hour at 37°C. Cells were seeded at 20,000/slide/well in 1 ml of SFMC and incubated in 5% CO_2_ at 37°C and 100% humidity. After 3 days growth, the cells were either treated with electric field 2 hours/day for two days in the incubator (37°C), or just put into the chamber without turning on the electric field (control group). After the 2^nd^ EF treatment, the cells were put into the cell differentiation medium that included 1% FBS in SFM. The medium was refreshed every 2 to 3 days. Cells were fixed in methanol at day 9 after differentiation and were stored at -20°C for immunocytochemistry staining. Multiple view field from each slide and multiple slides were chosen for the quantitative analysis. To ensure unbiased selection of the cells, view fields containing less than three cells were not selected for analysis. All experiments were repeated independently at least three times.

### 6. Immunocytochemistry staining

Fixed cells were washed 3 times for 5 minutes with PBS. Blocking was performed with Serum Blocking Solution (PBS contains 5% normal goat serum, 1% BSA, and 0.3% Triton X) at room temperature for 1 hour, and primary antibodies were added including mouse monoclonal anti-Nestin (1:200, Millipore), rabbit polyclonal anti-Glial Fibrillary Acidic Protein (GFAP, 1:400, Sigma), mouse monoclonal anti-beta-Tubulin Isotype III (1:800 Sigma)) and rabbit polyclonal anti-olig2 for oligodendroglial lineage cells (1:200, Millipore). Cells with primary antibodies were incubated at 4°C overnight.

The cells incubated with primary antibodies were washed 1 minute with PBS for 5 times, and secondary antibodies were added using goat Anti-Mouse IgG (Jackson ImmunoResearch Laboratories), or goat anti-rabbit (life technology Alexa fluor 488) at 1:200 with blocking solution at room temperature for 1 hour. Cells were washed 1 minute with PBS for 5 times. Nuclear counterstain was performed with DAPI (4',6-Diamidino-2-Phenylindole, Dilactate) at 1:1000 with blocking solution at room temperature for 15 min, washed 1 minute with PBS for 5 times, and mounted with Vectashield mounting media (Victor Laboratories). To avoid risk of cell loss, slides were sealed with nail polish (OPI RapiDry TopCoat).

### 7. Statistics

Throughout the text, mean ± standard error (SE) was reported. All data was verified for normal distribution and homogeneity of variance. Statistical significance was determined with unpaired t-tests, or one way repeated ANOVA followed by the post hoc Bonferroni test, using SigmaStat software (v. 3.0, Aspire Software International, Ashburn, VA). For the percentage comparison of cell differentiation with and without electric field, Chi-square test was used. Effects were considered statistically significant at p < 0.05.

## Results

### I. NPC culturing, stemness, and capability of differentiation

**Fig 3:**
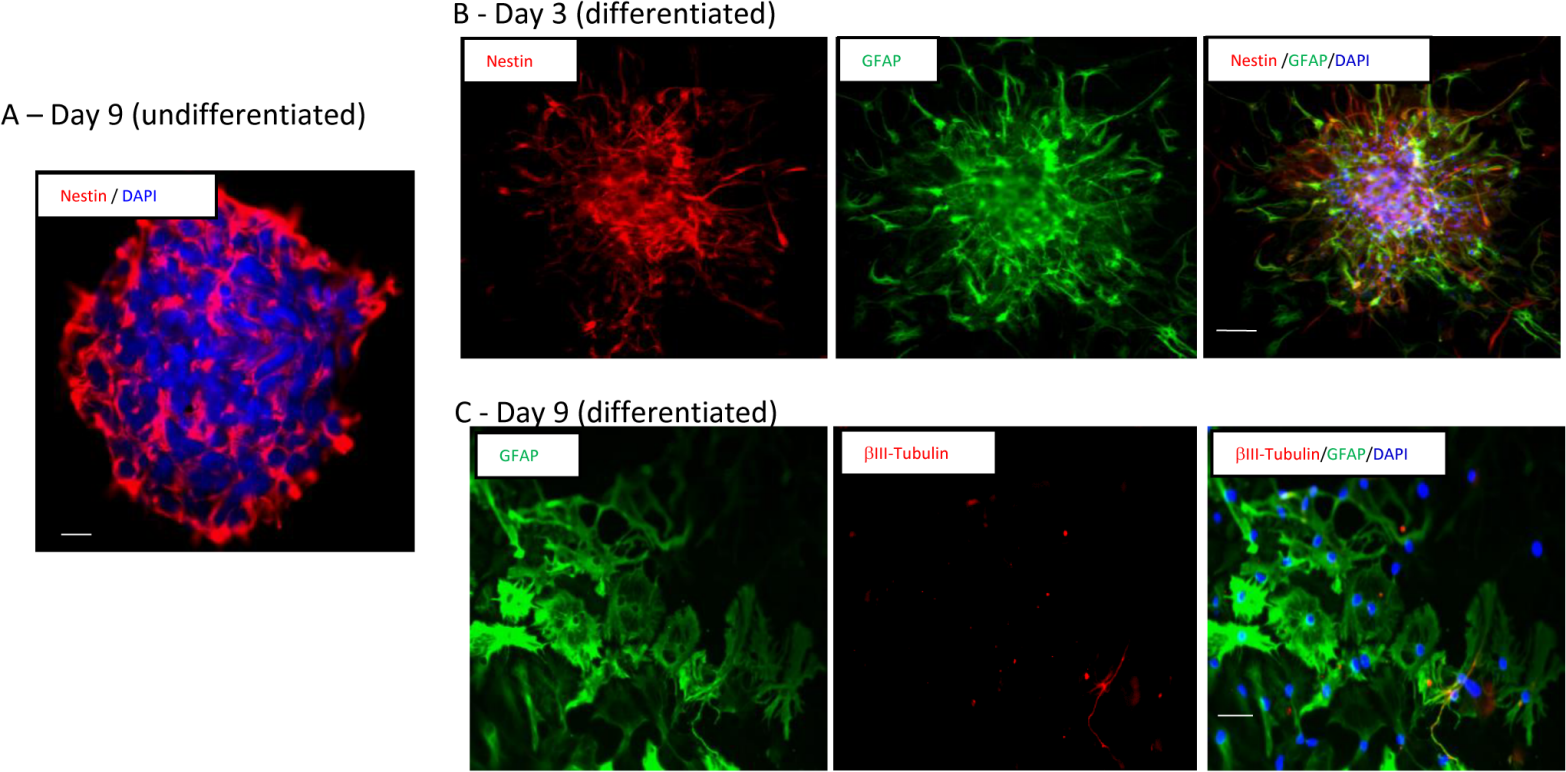
Stemness and differentiation of the cultured NPCs. (A) Neurospheres formed after 9 days growth of the isolated NPCs from the neonatal mice in the presence of EGF, bFGF, and heparin in serum free medium. Cells expressed neuronal stem cell marker Nestin (red). DAPI (blue) was used for nuclei and chromosome counterstain. (B) Partially differentiated neurosphere showing Nestin+ (Red) and GFAP+ (Green) after 3 days growth in serum free medium in the presence of 1% Fetal Bovine Serum (FBS). (C) 9 days after differentiation, cells largely became GFAP+ astrocytes (green), and a small amount of cells differentiated into βIII-tubulin+ neurons (red). Scale bar = 100 μm.

When cultured in the serum-free medium in the presence of EGF, bFGF, and heparin, NPCs formed neurospheres. Stemness and differentiation capability of the cultured NPCs were analyzed using common progenitor and neural lineage markers. GFAP was used as a marker of activated astroglia, differentiated neurons were labeled for βIII-tubulin, and DAPI (blue) was used for nuclei staining and the counting of the cells. When stemness was assessed at day 9 after growth in the serum-free medium with growth factor and heparin, the neurospheres from passages 3 to 4 expressed Nestin, a marker for neural stem/progenitor cells (Fig 3A). Cells dissociated from neurospheres and cultured in proliferative medium remained undifferentiated, with roundish and usually apolar morphology with Nestin immunofluorescence.

We withdrew the growth factors and replaced them with differentiating medium containing 1% fetal bovine serum in the serum-free medium. After 3 days in culture, the cells displayed a heterogeneous morphology suggesting that they were undergoing differentiation, evidenced by the loss of Nestin expression in comparison with neurospheres formed in SFMC, and the presence of the GFAP+ cells (Fig 3B). By the 9^th^ day, cells largely differentiated into GFAP+ astrocytes (59.4 ± 3.6%) because serum composition favors astrocyte specification [29]. A small amount of cells differentiated into βIII-tubulin+ neuronal lineage (8.2 ± 2.2%, Fig 3C) and Olig2+ oligodendroglial lineage cells (8.7 ± 0.6%). The percentage reported here is reflective of the results from previous publications [8, 29]. The GFAP+ cells showed multiple processes and a star-like shape. In contrast, βIII-tubulin+ cells exhibited the characteristics of multipolar neurons with two to five well-defined primary processes [27]. These results confirm the previous observations that NPCs have the ability to differentiate into multiple neural cell types *in vitro* [30, 31].

### 2. 115 V/m direct current (DC) electric field enhanced undifferentiated cell mobility and directional migration in a calcium-dependent manner

To explore the migration capability of NPCs in the presence of DC electric field and the potential role of calcium in mediating cell migration, we constructed a pair of Ag/AgCl electrodes in a culture dish and generated a 115 V/m DC electric field around the cells. Cell migration was video recorded for 1.5 hours and the initial and ending positions of the cells were recorded. We applied a grid mesh to quantify the cell movement. Cells that moved with a distance greater than 1 grid size (15 μm X 15 μm) in either horizontal or vertical directions were counted as mobile cells. Cells that failed to meet these guidelines were otherwise designated as immobile.

**Fig 4:**
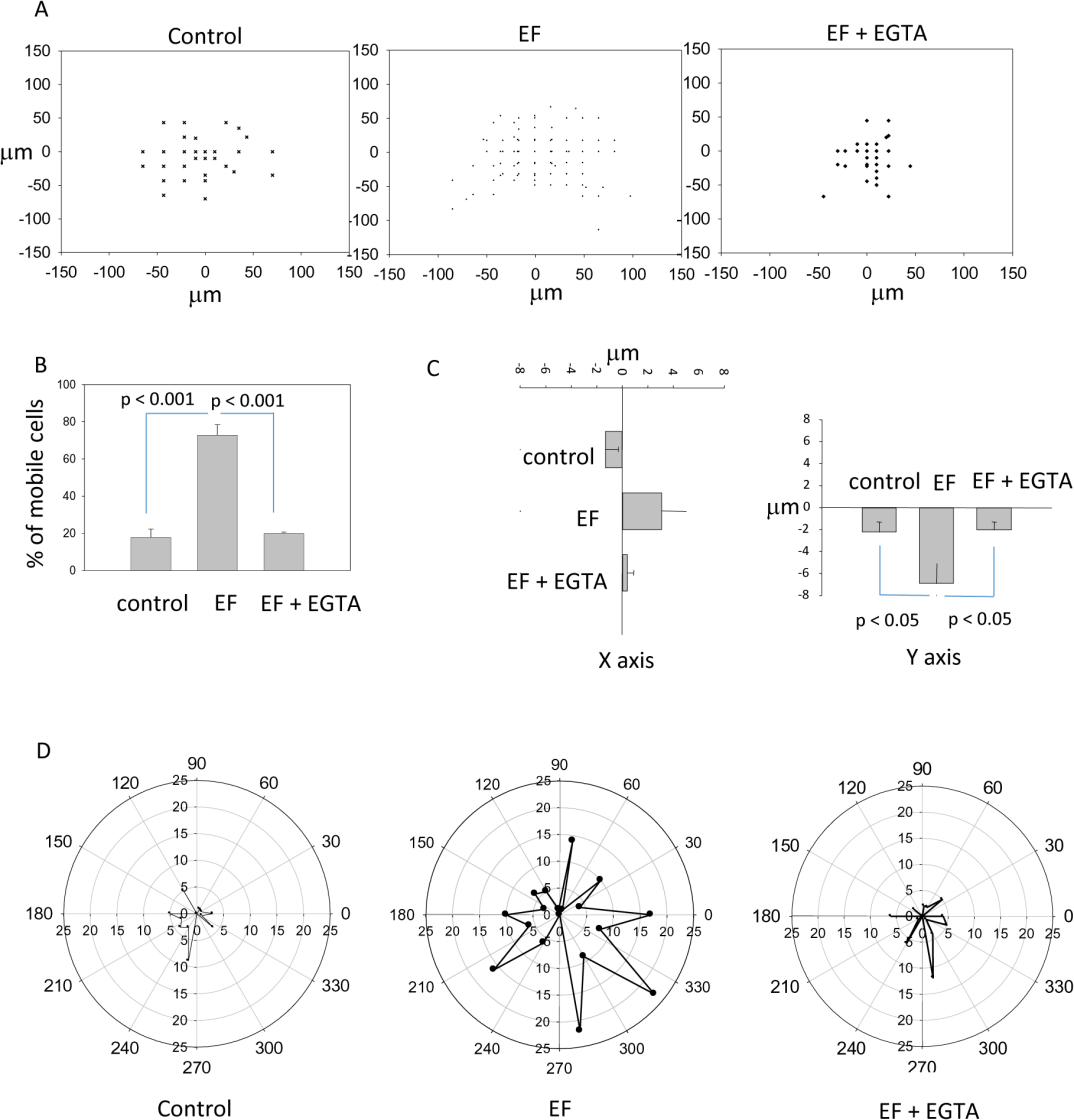
NPCs cultured in matrigel migrated in 115 V/m DC electric field (EF). (A) Kinematic analysis of cell migration in control, EF, and EF + EGTA groups. Distribution of cells at the end of the experiment in each experimental group. Plot here is the final relative location of cells assuming their original location is (x = 0; y = 0). (B) Percentage of mobile cells observed in each experimental group. (C) Distance of cell movement in horizontal (x) and vertical (y) directions after 90 minutes of electric field exposure. (D) Circular plots show the angle of cells’ movement in control, EF and EF + EGTA groups. The radii of these plots represent the number of cells migrated in that specific angle. Cells demonstrated clear cathodal migration in the EF group.

In the absence of DC electric field, cells demonstrated low likelihood of movement. Application of DC electric field to the cells greatly increased their mobility, however this did not happen in the EGTA treated cells. One way ANOVA revealed significant difference among the control, EF, and EF + EGTA groups (p < 0.001). In the non-EF treatment control, 17.79% ± 4.5% of the NPCs demonstrated movement in arbitrary directions. Presence of DC electric field significantly (p < 0.001) enhanced the cell’s migration capability (Fig 4A and 4B). DC electric field caused a total of 72.85% ± 5.6% of cells to migrate with a distance greater than a grid size. Presence of calcium chelator EGTA (I mM) in the medium significantly (p < 0.001) decreased the chance for cell’s migration in the field (20.3% ± 0.68%). There is no statistical difference in the likelihood of cell movement between the control group and the EF + EGTA treatment (p > 0.05). Taken together, 115 V/m DC electric field enhanced the migration capability of the cells through a calcium-dependent mechanism.

To quantify the amount of NPC movement in the electric field, we compared the mean distance that the migrated cells travelled in the three groups. One-way ANOVA revealed that the distance of movement in the field direction was significantly different among the three groups (p < 0.01). Overall, presence of DC electric field drove the cluster of cells to migrate to the cathode electrodes, and the mean amount of migration (6.9 ± 1.8 μm) is significantly greater (p < 0.05) than that measured from the control group (1.9 ± 0.9 μm). Addition of 1.0 mM EGTA to the medium prior to experimentation prevented the cells from cathodal migration (2.04 ± 0.73 μm). When direction that is perpendicular to the electric field is considered, the three groups are not significantly different (P > 0.05, one-way ANOVA) and cells did not show any preference of movement in this direction in the electric field (Fig 4C). To further illustrate the directional movement of the cells, we plotted the angle of the cells’ movement in a circular plot (Fig 4D) and found that large amounts of cells migrate to the cathodal direction in the electric field. In conclusion, cells migrated toward the cathode electrode in the DC electric field, and calcium chelator EGTA prevented cell migration under electric field implementation.

**Fig 5:**
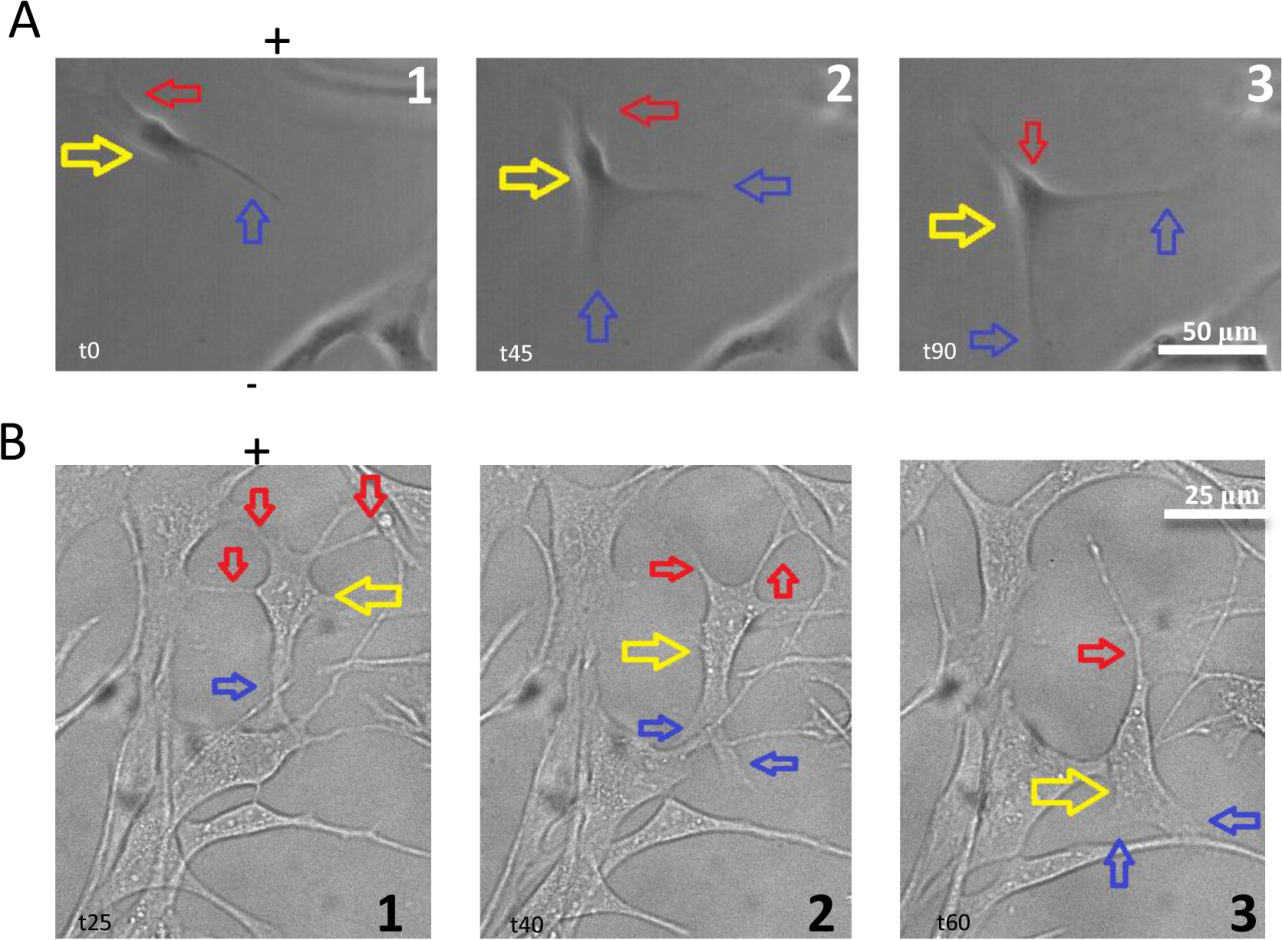
Cell morphology change during EF-directed migration. EF field was implemented for 90 min in each trial. Yellow arrows indicate movement of the cell body, red arrows for hind processes, and blue arrows for forward processes based on direction of EF field. Negative sign (-) indicates location of the cathode and the desired direction of migration. (A) One sample cell. (A1) At time = 0, one hind process (red) and one forward process (blue) are found while the cell body (yellow) remains evenly distributed and centralized. (A2) At t = 45 min, the hind process begins to detach while a new forward process forms in the cathodal direction; the cell body begins to redistribute in the direction of the forward processes. (A3) At time = 90 min, the hind process is absorbed by the cell body, the two forward processes have grown in size and length, and the cell body has moved in the cathodal direction. (B) A second sample cell. (B1) At time = 25 min, three hind processes are attached to nearby NPCs, anchoring the cell; one forward process is attached and the cell body is centralized. (B2) At t = 40 min, one hind process is detached from NPC (red), cell body has shifted downward (yellow), and one additional forward process has formed in the direction of the cathode (blue). (B3) At t = 60 min, all three hind processes have detached, the two forward processes have increased in size and length, and cell body has shifted further downward in direction of cathode.

**Fig 6:**
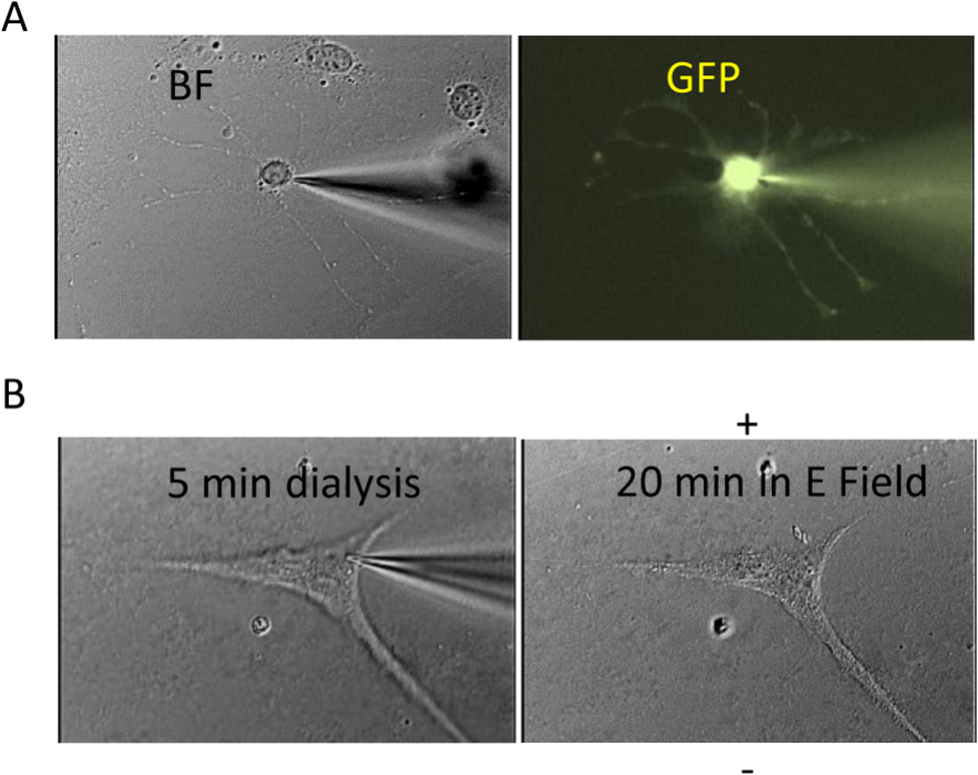
Intracellular administration of EGTA prevented the EF-mediated NPC migration. (A) An example of successful dialysis of fluorescence indicator Lucifer Yellow into the NPC via a patch pipette. (B) The cell was injected with EGTA (1 mM) with the patching pipette with 5 minutes of delivery before it was withdrawn. Consequent application of 115 V/m DC electric field for 20 minutes didn’t cause noticeable cell shape change and movement.

Multiple studies have reported the role of calcium in mediating cell shape change and movement [32]. To further examine cell morphology of NPCs under electric field stimulation, images were taken from EF treatment samples (Fig 5). We observed a clear cytoskeleton change when cells migrated. Migration of cells in the electric field is feathered by a “push and pull” movement, as characterized previously in amphibian neural crest cells [33] and several other cell types [34]. Fig 5 demonstrates two representative sequences of cell morphological change during migration inside the 115 V/m electric field. Marked by yellow arrows, the cell body moved towards the cathode (+), and its hind processes (red) follow. Blue arrows indicate the production and extension of new processes in the direction of cell migration. Both sample cells extended their processes facing the cathode electrode and withdrew those facing the anode electrode during cell migration. This type of cell motion in the electric field disappeared when EGTA was added to the medium (data not shown). We also applied EGTA (1 μM) intracellularly via the patching pipette. We found (using Lucifer Yellow as an indicator) that intracellular compounds would dialyze into the cell a couple of minutes after the cell was patched on (Fig 6A). After the EGTA was dialyzed into the cell with patching pipette (n = 8), we applied the 115 V/m electric field to the cell for 20 minutes and didn’t observe noticeable cell shape change or movement (Fig 6B).

### 3. 115 V/m DC electric field caused undifferentiated NPC membrane depolarization

To characterize the physiological properties of the cells under electric field exposure, we applied whole cell patch clamp recording to the cells that were seeded on the matrigel for 3–5 days. The undifferentiated cells grew processes and attached on the bottom within 2–3 days, and the resting membrane potential of the cells was found to be -57.3 ± 3.9 mV. To investigate the input resistance, cells were injected with 500 ms of step current via the patching electrode. NPCs had input resistance of 223.3 ± 36.4 MΩ. Cells depolarized with the patching electrode did not fire any action potential.

**Fig 7:**
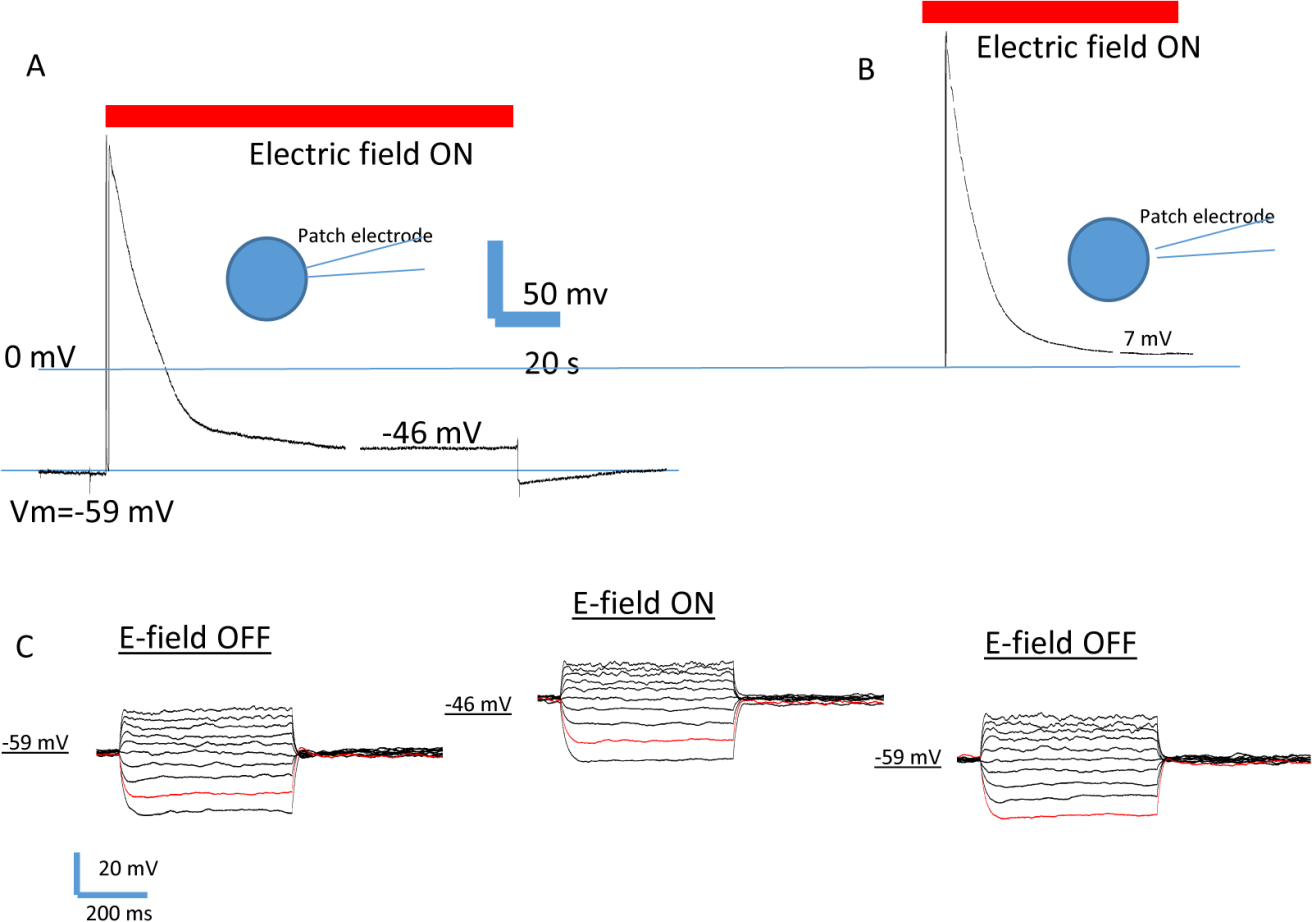
Depolarization of the cultured NPCs by 115 V/m DC electric field. (A) The patched cell had a resting membrane potential of -59 mV. When the DC electric field was turned on, the patching electrode recorded a large transient voltage change, which then stabilized after 1 minute at -46 mV. (B) To estimate the artifacts recorded by the electrode, after patching experiment, the same electrode was used to record the voltage changes (7 mV) at an isopotential line, next to the cell. This stimulus artifact was removed from the intracellular recording to obtain the resting membrane potential of the cell inside the E-field (- 53mV). Therefore, the cell was depolarized by 6 mV in the E-field. (C) I-V relations recorded from the cell before, during, and after DC electric field exposure.

We examined the effect of DC electric field on the membrane potential and input resistance by applying current clamp recording in the DC electric field. Fig 7 shows a typical recording obtained from a representative cell with resting membrane potential of -59 mV. When the DC electric field was turned on, it caused a large positive shift in the recorded potential, followed by a decay. This transition artifact lasted for about 1 minute before the patch electrode recorded a stable voltage of -46 mV. When the DC electrode field was withdrawn, the electrode recorded another transient artifact that lasted for about 40 seconds, and then reached a stable cell membrane potential of -59 mV (Fig 7A). The recorded shift of resting membrane potential, however, usually contains a field artifact that is imposed directly on the recording electrode [28]. After subtracting this stimulus artifact (Fig 7B), we determined that the cell was depolarized by 6.0 mV in the electric field. On average, we found cells were depolarized by 4.1 ± 0.4 mV (n = 8) in the electric field.

To compare the input resistance with and without electric field exposure, we applied step current to depolarize and hyperpolarize the cell membrane, and recorded the I-V relations during current injection. Fig 7C shows a typical recording that contains I-O traces before, during E-field application, and after the E-field was turned off. We did not find significant changes in the input resistance during electric field exposure (p > 0.05, paired t-test). We constrained our experiments to be less than 5 minutes since longer time recording was impractical due to the migration of the cell away from the electrodes and the subsequent rupture of the cell membrane.

**Fig 8:**
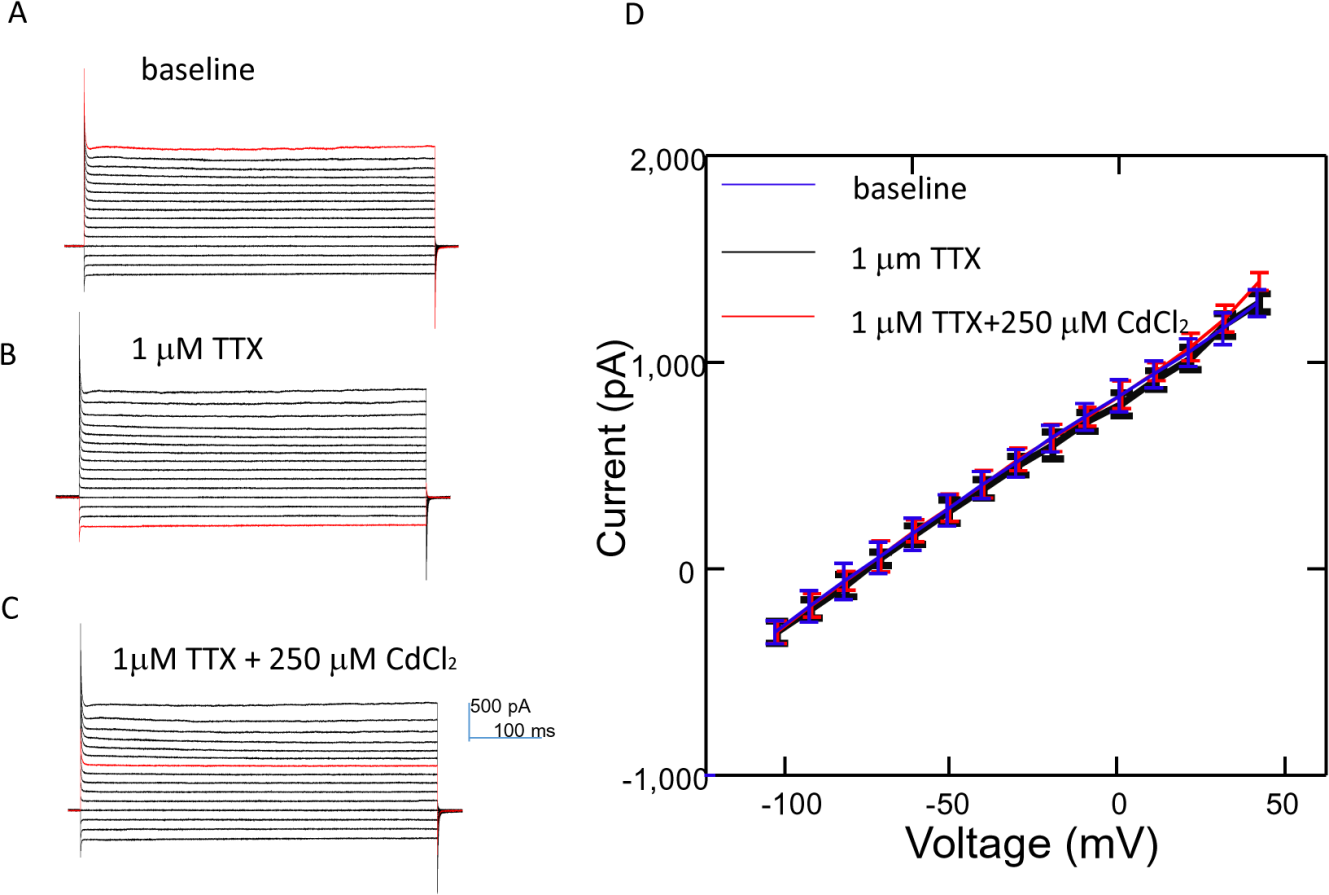
The undifferentiated NPCs did not express voltage-dependent calcium channels. (A) Voltage clamp recording. Cells were held at various potentials and the corresponding currents were recorded. (B) TTX (1.0 μM) was applied into the medium to block Na channels. (C) Both TTX (1.0 μM) and CdCl_2_ (250 μM) in the medium. No existing evidence demonstrates the inward current that corresponds to either Na^+^ or CdCl_2_ sensitive channels (VDCCs). (D) Current-voltage relationship.

Since the buffering of medium calcium prevented cell migration in the electric field, and cells depolarized in the electric field, we question whether cell migration in EF was mediated by the calcium influx via voltage-dependent calcium channels (VDCCs). However, in voltage clamp recordings, we did not observe inward current that corresponded with the depolarizing step currents. Instead, cell membranes illustrated leakage current in response to various clamping voltages. To eliminate the possibility that depolarization–induced Na^+^ current will bias the recording, we applied TTX (1.0 μM) into the media, and found no evidence of Na* current. Further addition of non-selective VDCC blocker CdCl_2_ (250 μM) into the medium did not yield significant changes in inward current (p > 0.05), further confirming the absence of depolarization-mediated calcium influx (Fig 8). We conclude that calcium entry into the cell during electric exposure is not via VDCCs.

### 4. 115 V/m DC electric field favored NPC differentiation into neurons with no impact on cell morphology

Membrane depolarization and calcium entry have been reported to play an important role in neural differentiation of stem cells. For example, optogenetic stimulation causes membrane depolarization and rapid differentiation of induced pluripotent stem cells into neurons [35]. Influx of calcium has been shown to guide cells toward neurons during differentiation [27]. We, therefore, used double and triple staining in immunocytochemistry to investigate the impact of DC electric field exposure on cell differentiation in the differentiation medium. Immediately after a 2-day electric field exposure protocol (2 hours/day), cells were allowed to differentiate for a total of 9 days in the differentiation medium. Cell fate after differentiation was then analyzed using several different cell markers. Using DAPI as the cell nuclear marker, a total of 1639 cells in the control (non-EF) groups, and a total of 1663 cells in the experimental (EF group) were analyzed. GFAP and βIII-tubulin were used to detect astrocytes and neurons, respectively. Olig2 was used for oligodendroglial lineage cells. Nestin was used to track the undifferentiated neuronal stem cells.

**Fig 9:**
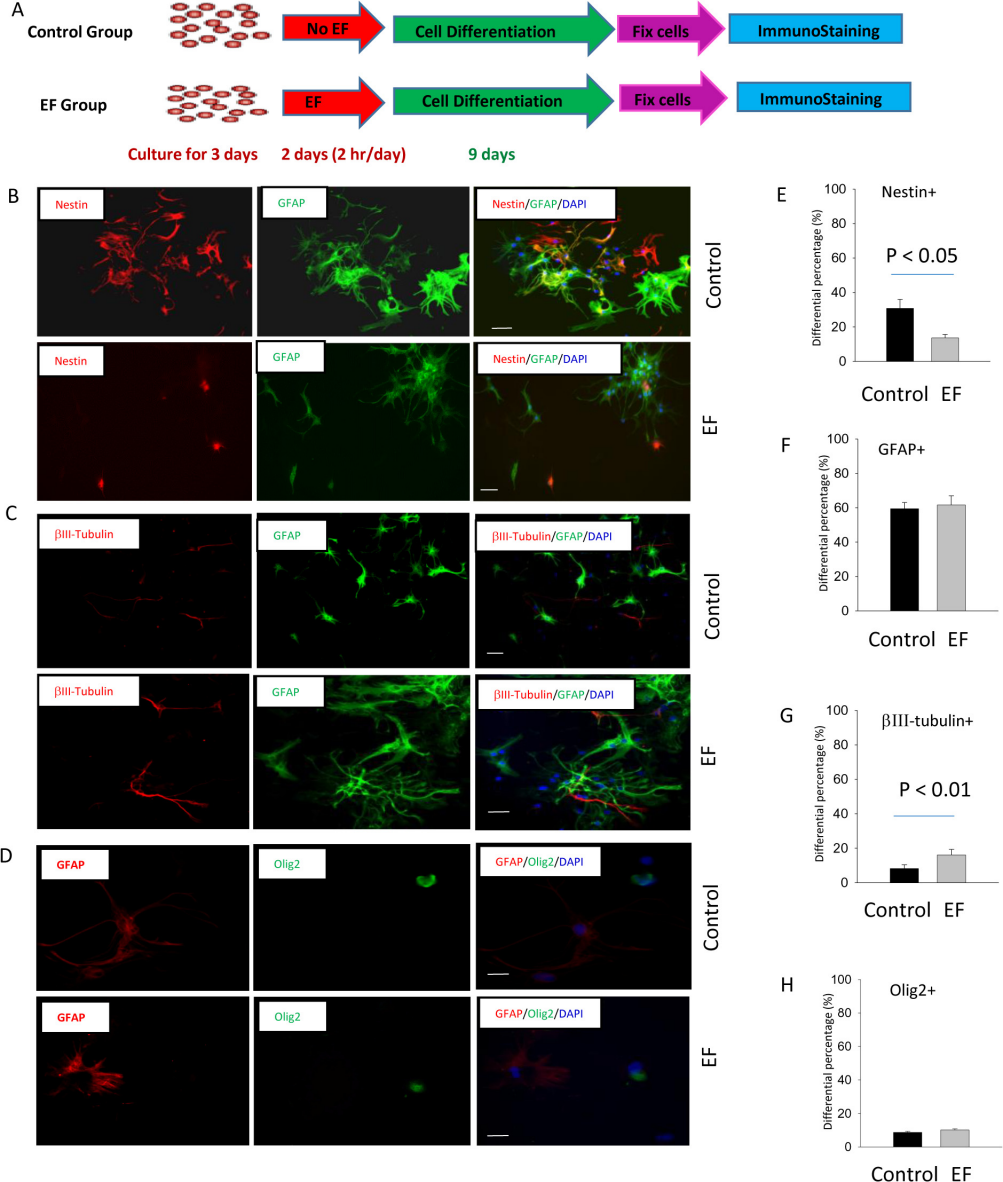
115 V/m DC electric field enhanced the neuronal differentiation. (A) Protocol for electric field exposure and cell differentiation. Undifferentiated cells were exposed in the electric field 2 hours/day for two days, and cells were cultured in the serum free medium in the presence of 1% fetal bovine serum (FBS). Cell differentiation assay was performed 9 days after NPC growth in the differentiation medium. (B) Double staining of Nestin+ and GFAP+ cells in the control (non-EF) and experimental (EF) groups. (C) Double staining of βIII-tubulin+ and GFAP+ cells in the control (non-EF) group and EF groups. (D) Double staining of GFAP+ and Olig2+ cells in the control (non-EF) group and EF groups (E-H) Statistical summary for the percentage of cell differentiation into different cell types (E: Nestin+; F: GFAP+; G: βIII-tubulin+; H: Olig2+) with and without electric field exposure. Scale bars = 100 μm.

Electric field exposure significantly enhanced the percentage of differentiated cells (Fig 9). In the control group, we observed that 30.8% ± 5.2% cells were Nestin+ after 9 days of differentiation. In contrast, a significantly (p < 0.05) lower percentage of Nestin+ cells (13.6% ± 2.0%) were observed in EF exposure group. Among the differentiated cells, we observed a significant increase in the expression of neuronal marker βIII-tubulin in 9 days. In control, 8.2% ± 2.2% cells were βIII-tubulin+ neurons. In the EF group, 16.9 ± 5.3% were βIII-tubulin positive (p < 0.01). EF did not affect the percentage of astrocytes in the overall cell population. After EF exposure, 61.6% ± 2.7% of the cells became GFAP positive astrocytes, which is not statistically different from that in the control group (59.4% ± 3.6%, p > 0.05). F did not affect the percentage of oligodendrocyte either. After EF exposure, 10.1% ± 0.7% of the cells became Olig2 positive astrocytes, which is not statistically different from that in the control group (8.7% ± 0.6%, p > 0.05).

**Fig 10:**
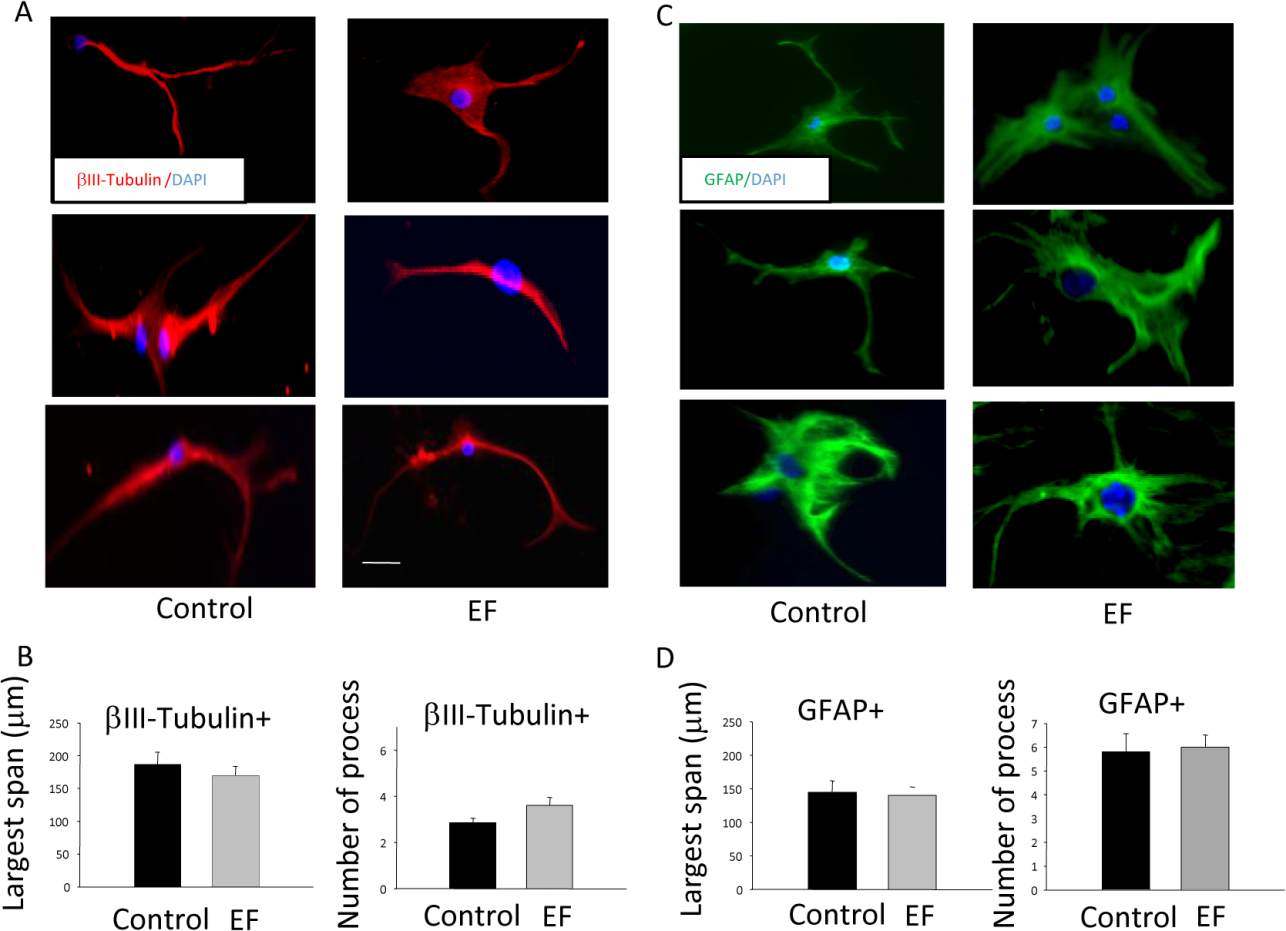
115 V/m electric field did not affect the morphology of the differentiated cells. Maximal span of the cell and the number of processes were measured. (A) Examples of βIII-Tubulin+ neurons in the control and EF exposed groups. (B) Statistics show that the largest span of the cells and number of processes in βIII-Tubulin+ were not significantly altered by EF exposure. (C) Example of GFAP+ cells in the control and EF exposed groups. (D) Statistics show that the largest span of the cell and the number of processes in the GFAP+ cells were not significantly altered by EF exposure. Scale bars = 50 μm.

Immunostaining also allowed us to further quantify morphology of the differentiated cells. Specifically, we measured the largest span of the differentiated cell as an indicator of its size [36]. We also measured the number of processes of the differentiated cells, a parameter that has been used to distinguish NPC-derived neurons and astrocytes [27]. After EF exposure, the βIII-tubulin+ neurons spanned with an average of 186.9 ± 18.7 μm, which is not different from the control group (169.9 ± 13.9 μm, p = 0.47). In the EF group, neurons contain 3.6 ± 0.35 processes, which is not significantly different from those in the control group (Mann-Whitney Rank Sum Test, p = 0.729). Similarly, when morphology of GFAP+ cells are compared, we didn’t observe significant difference in cell size (144.7 ± 17.4 μm in control vs 140.0 + 12.3 μm in EF group, p > 0.05) or number of processes (5.8 ± 0.77 in control vs 6.0 ± 0.51 in EF group, p > 0.05). Even through exposure of NPCs to electric field skewed the cells for neuronal differentiation, it did not cause significant morphological changes among the several tracked, differentiated cell types (Fig 10).

## Discussion

Although DC and AC electric field have been reported to be used for cell migration in various stem cell types, the mechanisms are still largely unknown. Using multi-dimentional approaches, including intra- and extracellular calcium buffering and live cell imaging, we report that a 115 V/m DC electric field can enhance mobility and cause cathodal migration in the cultured NPCs via a calcium-mediated mechanism. In addition, using the combined tools of whole cell patching and pharmacology, we found that NPCs are depolarized in the electric field. We exclude the possibility that calcium entry is due to VDCCs, and attribute it to the depolarization-induced passive leakage. These studies provide further insights for the cellular mechanism of NPC migration in the DC electric field. Furthermore, post-stimulus analysis indicated that the 115 V/m DC electric field enhanced cell differentiation and increased the percentage of neurons in the differentiated cell population. These findings are the first of its kind to report a field intensity that can apply both cell migration and differentiation control.

### Selection of 115 V/m DC electric field

We did not explicitly explore the question of whether 115V/m is the optimal intensity for both migration and differentiation processes since this may have involved large-scale live cell imaging, IHC staining, and cell counting experiments under various stimulus intensities. We chose this field intensity based on literature review, our preliminary observations, and theoretical analysis. In literature, both higher and lower intensity of DC electric fields have been explored for the control of stem cell migration and differentiation. For example, Babona-Pilipos used 250 V/m for the migration of adult neural stem cells [14]. A recent study by Meng et al [15] showed that adult hippocampal cell line-derived NPCs (HCN-A94 cells) exhibited cathodal galvanotaxis in the presence of 250 V/m and 500 V/m DC fields. A major concern for the high intensity field is cell viability. For example, when neural stem/progenitor cells from hippocampus were exposed to 437 V/m DC EF, decrease in cell viability was observed [26]. When the authors used a much lower (46 V/m) field, the field was too weak to induce any changes in NPC differentiation. In agreement with these studies, our preliminary data showed that intensity two times higher than 115 V/m would quickly damage the cells extracted from the P2-7 mice in our experiments, and field strength much lower than 115 V/m did not induce observable migration. We also theoretically estimated the amount of polarization (see later discussion, and also [37]) that DC electric field can produce on the cell membrane before the patch-clamp experiment, and found that to generate several mV of depolarization (recordable by patch electrode and possiblely meaningful physiologically), 115 V/m is an appropriate selection. We therefore restricted our study to the single intensity of 115 V/m, which was able to generate both observable cell migration and membrane depolarization, as well as provide significant effects in guiding cell differentiation into neurons.

### Membrane polarization and calcium-mediated NPC migration in the E-field

Our results show that NPC mobility was greatly enhanced in the 115V/m electric field towards the cathodal electrode. This intensity is larger than the endogenous EF (31.8 V/m) recorded from the explants of the subventricular zone, which is responsible for the chain migration of the SVZ explants in 3D culture [38]. In our experiments, the amount of migration is less than that reported previously for the same cell in the DC electric field [14], possibly due to a weaker field intensity and shorter field exposure time in our experiments (to avoid potential cell death). The migration is likely mediated by the cytoskeleton changes, with the posterior part of the cell contracting and the anterior of the cells extending (Fig 5). These results support the hypothesis that cytoskeletal changes play a significant role in mediating cell migration in the electric field. This hypothesis believes that the elongation of actin filaments, usually at the leading edge of the cell [39], is the main driving force for cell movement.

Our results suggest the involvement of calcium in electric field-mediated NPC migration. It has been shown previously that acute exposure of human adipose-derived stem cells (hASCs) to alternating current (AC) sinusoidal electric fields of 1Hz induced a dose-dependent increase in cytoplasmic calcium [40]. Calcium-mediated cytoskeleton change is tightly correlated with the calcium-mediated polymerization of actin. It is believed that a reduction in [Ca^2+^]_i_ could promote polymerization of actin, thereby causing protrusion of the cell [41]. Although the mechanisms responsible for actin dynamics at the rear of migrating cells are still not well understood, [Ca^2+^]_i_ might be increased, resulting in actin depolymerization [42] [43] and contraction of this side of the cell, which then propels the cell towards the cathode [34].

The pathways for calcium entry into the cytoplasm during electric stimulation are unclear. There are at least several possibilities including activation of voltage-dependent channels, calcium release from the internal organelle, or by passive influx from the extracellular space. Recent evidence confirms that changes in plasma membrane potential is contingent upon the migration capability of the dictyostelium cells [44]. Accordingly, we have recorded a slight depolarization of the cell membrane in the DC electric field (Fig 7). The amount of depolarization measured by the patch pipette agrees with the theoretically predicated *Vm* = 1.5*Edcos*(*θ*), where E is the intensity of the field, d is the diameter of the cell, and cos(*θ*) is a geometrical factor [37]. If we assume d = 40μm as the cell diameter, we obtained a theoretical predication of 6.9 mV of depolarization, a measure close to what we obtained in whole cell patching experiments. However, evidence favoring the presence of voltage dependent calcium channels (VDCCs) in the undifferentiated NPCs is lacking, and the amount of E-field induced depolarization is insufficient in activating the VDCC [27], which makes this an unlikely calcium pathway for entry. In supporting this notation, we directly test the presence of VDCC by measuring inward current with and without the VDCC blocker CdCl_2_ (Fig 8), but found no evidence of VDCC-mediated calcium influx.

Alternatively, mitochondria play a role in regulating (buffering) physiological Ca^2+^ levels that function with a fast uptake component [45]. Nanosecond-pulsed electric fields (nsPEFs) were shown to affect the mitochondrial membrane [46, 47] and cause calcium release from internal stores [48]. Although little is known about the effects of the DC electric field on internal organelles such as mitochondria, theoretical works have shown that the cytoplasmic membrane could provide “shielding effects” against the DC electric field, and prevent the electric field from penetrating into the cell [49].

Therefore, we tend to accept that the passive calcium influx through the leaked membrane into the cell might cause the dynamic changes in the calcium homeostasis inside the cell. Our patch data illustrated a leakage current that was associated with the cell depolarization (Fig 8). As a cation, calcium could enter the cell membrane from the anode side and cause the local increase of the calcium level, resulting in actin depolymerization and contraction of the cell membrane. In contrast, calcium leaves the cell membrane from the cathodal side, introducing local decrease of calcium, actin polymerization, and membrane extension. Future endeavors will be focused on combined intracellular imaging and live cell migration imaging in the electric field to further link the intracellular calcium signals with the cytoskeleton changes in its migration.

### Post-stimulus neuronal differentiation

NPC differentiation depends on the type of electric field (i.e., AC vs. DC), its magnitude, and its frequency [50]. It appears that DC electric field is more efficient in guiding the cells into neurons. We found that the 115 V/m DC electric field skewed the differentiation profile in favor of neurons (Fig 9). A tripled intensity (437 V/m) DC electric field has also shown effectiveness in promoting neuronal differentiation (associated with decreases in cell viability), but not oligodendrocytes or astrocytes [26]. In contrast, a 46 V/m alternating current EF (1 Hz) enhanced the propensity of astrocyte differentiation over neuronal differentiation of NPCs [26]. Taken together, these data establish the key role of systematic delivery of DC electric stimuli as guidance cues in promoting neural-like differentiation. Future work will attempt to increase DC field intensity to maximize both cell migration and neuronal differentiation capability, and maintain minimal damage to the cells.

Our data begin to establish a link between calcium entry, depolarization, and cell differentiation. Ca^2+^ concentration has been reported to be related to field-induced modification of differentiation. For example, static magnetic field enhanced the potential of bone marrow stem cells to differentiate into primordial germ cells [51]. Ca^2+^ ion cyclotron energy resonance may drive cardiac-specific differentiation in human adult cardiac progenitor cells [52].

For neural stem cell differentiation, it appears that electric field could also promote neuronal differentiation via calcium influx. Extremely low-frequency (50 Hz) electromagnetic fields promoted neuronal differentiation of neural stem cells *in vitro* by up-regulating Ca(v)1 channel activity [53]. In the same cell type that we studied, previous literature has already established that its differentiation into neurons is mediated by calcium [27]. The authors found that during differentiation, influx of the calcium is via the newly formed voltage-dependent calcium channels. Cells were cultured in differentiating medium with either nifedipine or the L-channel activator Bay K 8644. The latter Bay K 8644 treatment significantly increased the percentage of β-III-tubulin+⁄MAP2+ cells, whereas nifedipine produced opposite effects. Although VDCCs were not expressed in the undifferentiated cells (Fig 8), we believe DC field may cause calcium influx via passive diffusion, due to membrane depolarization, which promotes neuron differentiation. Therefore, our results further extend previous work by showing that electric field, via calcium signal promotion at the beginning of the differentiation, is sufficient to guide the cell differentiation in favor of neurons (Fig 9). It will be interesting to monitor calcium levels post-stimulus, and relate the differentiation profile with the dynamic changes of calcium to further clarify the role of field-induced calcium signaling in simultaneous control of cell migration and differentiation.

## Conclusions

The identification of neural stem cells in the adult brain has led to the development of endogenous neural precursor cell activation as a method to repair the injured CNS [1, 54, 55]. Critical to the success of such self-repair schemes is the effective expansion and recruitment of stem cells into the sites of injury or disease, and subsequent differentiation into desired cell types. This study reports a DC field intensity (115 V/m) that is capable of controlling both aspects of the cell fate. We believe this finding is a significant contribution to researchers interested in simultaneous control of cell’s migration and differentiation with optimized field properties. We suggest that harnessing the migratory and differentiation potential of NPCs in the presence of an electric field *in vivo* may provide a means to enhance endogenous neural repair and tissue regeneration. This study will pave a way for our future clinical aim to combine stem cell therapy and electric stimulation in the treatment of neurological diseases.
